# Updated Search for Alternative Unit Cells

**DOI:** 10.1063/4.0000890

**Published:** 2025-10-27

**Authors:** Herbert J Bernstein, Lawrence C Andrews

**Affiliations:** 1 Fresh Pond Research Institute, Cambridge, MA, USA; 2 Ronin Institute for Independent Scholarship, Kirkland, WA, USA

## Abstract

Since 2014 SAUC^1^ has provided a search engine to find crystallographic unit cells similar to a given probe cell. The data for the version available on the LRL_WEB, Lattice Representation Library Tool Web Page^2^, has been updated to report on 191,000 Protein Data Bank macromolecular unit cells and 434,000 small molecule unit cells from the Crystallography Open Database for a searchable database of over 625,000 small molecule and macromolecular unit cells. The update was done in February 2025. The search copes well with complex boundary cases and will be updated approximately semi-annually.

A sample of a search for the cells in the vicinity of the chymotrypsinogen cell from PDB entry 7KTY^3^ is attached.
